# 矮小同源盒基因启动子区域甲基化诊断肺癌价值的*meta*分析

**DOI:** 10.3779/j.issn.1009-3419.2021.101.27

**Published:** 2021-07-20

**Authors:** 强 刘, 帅 王, 国田 裴, 影顺 杨, 宇清 黄

**Affiliations:** 100080 北京，北京市海淀医院胸外科 Department of Thoracic Surgery, Haidian Hospital, Beijing 100080, China

**Keywords:** 肺肿瘤, *SHOX2*基因, 启动子, 甲基化, *Meta*分析, Lung neoplasms, *SHOX2* gene, Promoter, Methylation, *Meta*-analysis

## Abstract

**背景与目的:**

肺癌是临床上最为常见的恶性肿瘤，晚期患者预后差，5年生存率低，因此早期诊断成为提高患者预后的关键。近年来随着分子生物学技术的发展，一些关键驱动基因的异常修饰如甲基化成为肺癌早期诊断的重要方法。本研究旨在采用循证医学方法量化评价矮小同源盒基因（short stature homeobox 2, *SHOX2*）启动子区域异常高甲基化诊断肺癌价值。

**方法:**

系统检索MEDLINE、EMBASE、Ovid、Web of Science、中国期刊全文数据库（CNKI）中涉及*SHOX2*基因启动子区域甲基化与肺癌关系的相关文献，并根据纳入与排除标准进行文献筛选。提取原始研究中*SHOX2*启动子甲基化相关数据，计算以SHOX2启动子甲基化为参考诊断肺癌的敏感性、特异性及受试者工作特征曲线（receiver operating characteristic curve, ROC）下面积。

**结果:**

最终纳入本次*meta*分析的文献13篇，各项研究间存在明显的统计学异质性（*P* < 0.05），数据合并均采用随机效应模型。*SHOX2*基因启动子甲基化诊断肺癌的敏感性为0.75（95%CI: 0.74-0.77）、特异性为0.89（95%CI: 0.88-0.91）；阳性预测值为6.75（4.56-9.99），阴性预测值为0.36（0.25-0.52）；诊断优势比为23.16（11.34-47.31），ROC曲线下面积为0.9。

**结论:**

*SHOX2*基因启动子高甲基化在肺癌患者血清、支气管灌洗液和胸腔积液中发生率均较高，可作为辅助诊断肺癌的生物学标志物。

## Abstract (en)

**Background and objective:**

Lung cancer is the most common malignant tumor in clinic. The prognosis of advanced patients is poor, and the 5-year survival rate is low. Therefore, early diagnosis becomes the key to improve the prognosis of patients. In recent years, with the development of molecular biology technology, aberrant modification of some driver genes, such as methylation, has become an important method for early diagnosis of lung cancer. The purpose of the present work was to quantitatively evaluate the diagnostic value of abnormal hypermethylation in short state homeobox 2 (*SHOX2*) promoter region in lung cancer by evidence-based medicine.

**Methods:**

We searched MEDLINE, EMBASE, Ovid, Web of Science and CNKI for literatures related to the relationship between *SHOX2* gene promoter hypermethylation and lung cancer. The data of *SHOX2* promoter hymethylation in the original study were extracted. The diagnostic sensitivity, specificity and area under receiver operating characteristic (ROC) curve of *SHOX2* promoter methylation were calculated.

**Results:**

Finally, 13 publications were included in this *meta*-analysis, and due to significant statistical heterogeneity among the studies (*P* < 0.05) the data was pooled by random effect model. The diagnostic sensitivity and specificity of *SHOX2* promoter hypermethylation in the diagnosis of lung cancer were 0.75 (95%CI: 0.74-0.77) and 0.89 (95%CI: 0.88-0.91), respectively; The positive likelihood ratio value was 6.75 (4.56-9.99), and the negative predictive value was 0.36 (0.25-0.52); The diagnostic odds ratio was 23.16 (11.34-47.31), and the area under the ROC curve was 0.9.

**Conclusion:**

*SHOX2* gene promoter hypermethylation is high in serum, broncholavage fluid and pleural effusion of lung cancer patients, which can be used as a biomarker for auxiliary diagnosis of lung cancer.

肺癌是临床上最为常见的恶性肿瘤，也是男性恶性肿瘤死亡的第一位。流行病学数据^[1]^显示，2021年北美预测肺癌新发病例为235, 760例，死亡131, 880例。肺癌已成为发病率和死亡率最高的恶性肿瘤^[2]^。早期肺癌患者进行以手术为主的综合治疗预后较好，而晚期肺癌预后差，5年生存率低。因此，对肺癌进行早期准确的诊断十分重要^[3]^。

矮小同源框2（short stature homeobox 2, SHOX2）也称为同源框蛋白Og12X或配对相关同源框蛋白SHOT基因，*SHOX2*基因编码的蛋白是同源框基因家族的一员，该家族编码含有代表DNA结合域的60个氨基酸残基。同源框蛋白被认为在生物体内广泛表达的转录调节因子，具有复杂的生物功能。近年来有研究^[4]^报道，*SHOX2*基因启动子区域异常高甲基化与肺癌的发生发展有关，该基因异常高甲基化可能发生在肺癌早期并与高甲基化后该基因的失活有关。陆续有研究报道了*SHOX2*基因启动子高甲基化发生频率在肺癌患者与正常对照人群的发生情况；有研究^[4]^显示，肺癌患者血清等组织中*SHOX2*基因启动子高甲基化发生率高于对照组人群。但关于*SHOX2*基因启动子高甲基化作为肺癌患者诊断生物学标志物的可行性研究鲜有道报。本研究采用循证医学的方法量化评价*SHOX2*启动子区域甲基化诊断肺癌价值。

## 1 材料与方法

### 1.1 文献检索

系统检索Medline、EMBASE、Ovid、Web of Science、中国期刊全文数据库（CNKI）中涉及*SHOX2*基因启动子区域甲基化与肺癌关系的相关文献，检索语种为英语和汉语。以“SHOX2”、“Short stature homeobox 2”、“OG12”、“SHOT”、“OG12X”、“non-small cell lung cancer”、“lung cancer”、“lung neoplasm”为自由词，检索Medline、EMBASE、Ovid、Web of Science等英文数据库；以“肺癌”、“肺肿瘤”、“非小细胞”、“矮小同源盒基因基因”、“SHOX2”为关键词或题名检索CNKI中文数据库。同时，我们对已纳入的研究的参考文献进行进一步评估，以发现可能符合要求的研究。

### 1.2 入选标准

① 研究设计：临床病例对照、队列研究或观察性研究；②研究对象：肺癌患者经病理学或细胞学明确确诊；③检测方法：组织标本中*SHOX2*基因启动子甲基化水平采用甲基化特异性PCR（methylation specific PCR, MSP）方法检测；④结果：原始研究中给出或可间接计算出肺癌患者和对照患者各个组织标本中的*SHOX2*基因启动子甲基化频率。

### 1.3 数据提取

两位研究者刘强、王帅对纳入的原始研究进行分别阅读提取数据和基本信息，提取的内容包括：纳入研究的一般情况，包括第一作者、国家、发表时间、杂志名称；纳入研究的基本特征，包括原始研究中肺癌患者及对照组的人种、样本量、甲基化检测方法；原始研究结果部分指标，包括原始研究中给出或可间接计算出肺癌患者和对照患者各个组织标本中的*SHOX2*基因启动子甲基化频率。

### 1.4 文献质量评价

以观察性流行病学研究报告规范（strengthening the Reporting of Observational Studies in Epidemiology, STROBE）中所列举出的必需项目清单为标准，依次对纳入研究的题目、摘要、前言、方法、结果、讨论6个部分22个项目进行质量评价，每个项目1分，满分为22分。

### 1.5 统计学方法

数据采用meta-DiSc1.4（http://www.biomedsearch.com）统计软件进行分析。在汇集数据之前各个研究间的统计学异质性采用*I*^2^检验，当*I*^2^ > 50%时，认为存在统计学异质性采用随机效应模型，反之采用固定效应模型进行数据合并，*P* < 0.05为存在统计学差异。

## 2 结果

### 2.1 检索结果

检索相关数据库初步获得868篇相关文献，首先经过查重，剔除重复发表文献44篇，通过阅读标题和摘要剔除明显不符合要求文献800篇，最后通过阅读全文剔除不符合要求的文献11篇，最终纳入本次*meta*分析的文献13篇^[5-17]^（图 1），13篇文献的基本情况见表 1。

**图 1:**
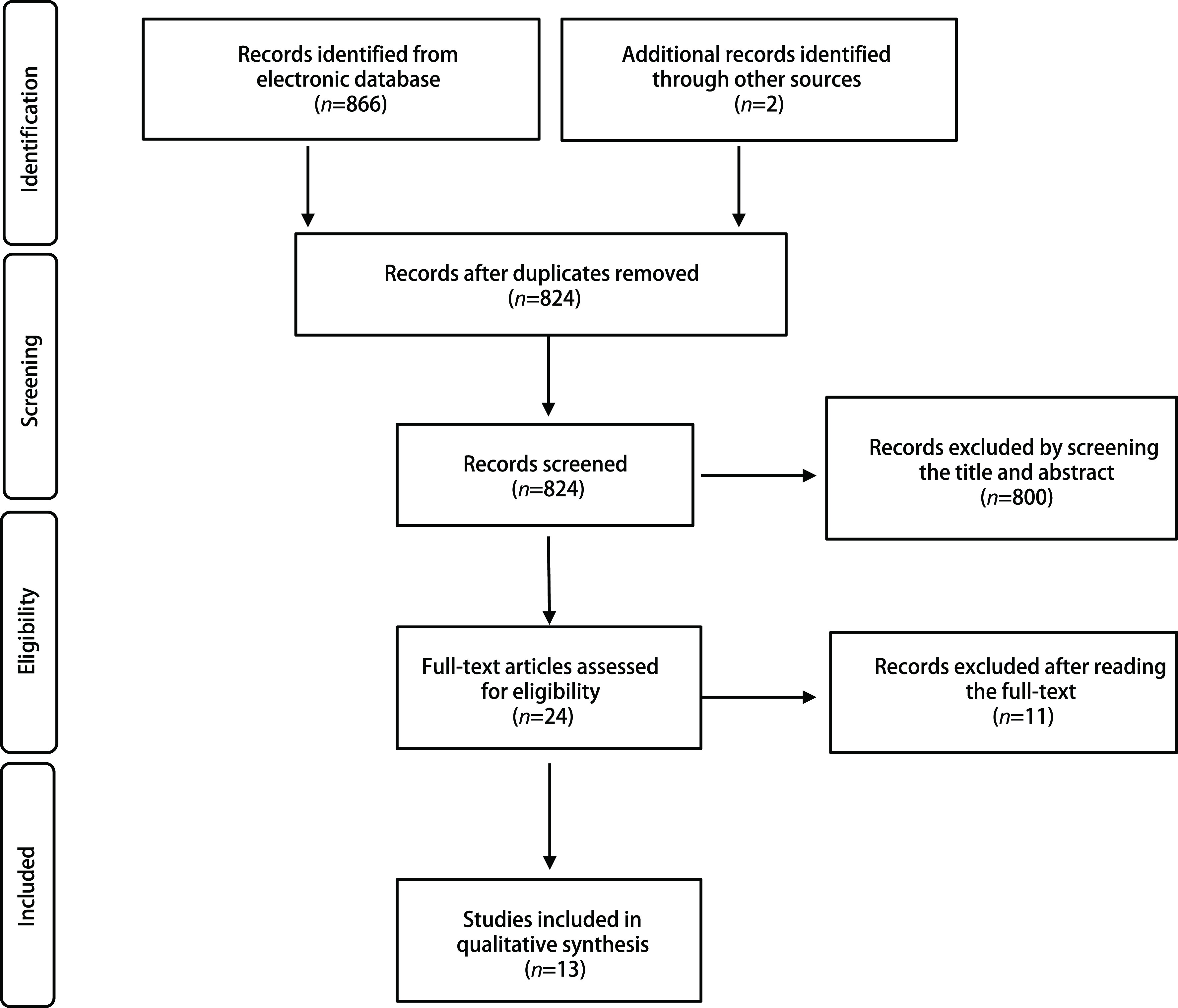
文献检索及纳入与排除相关研究流程图 Flow chart of literature retrieval according to inclusion and exclusion criteria

**表 1:** 纳入研究的基本特征 General characteristics of the included studies

Author	Time	Sample size	Cancer	Control	TNM	Tissue	Country	STROBE
			(M+/M-)	(M+/M-)				
Wang N^[5]^	2018	120	57/23	1/39	Ⅰ-Ⅳ	Serum	China	9
Rong QP^[6]^	2018	48	18/20	2/8	Ⅲ/Ⅳ	Serum	China	11
Zhang YM^[7]^	2016	277	98/32	34/113	NA	BLAF	China	9
Ren M^[8]^	2017	253	79/44	10/120	Ⅰ-Ⅳ	BLAF	China	14
Schmidt B^[9]^	2010	523	190/91	12/230	Ⅰ-Ⅳ	BLAF	German	12
Kneip C^[10]^	2011	343	112/76	16/139	Ⅰ-Ⅳ	Serum	German	14
Dietrich D^[11]^	2012	204	78/22	4/100	Ⅰ-Ⅳ	BLAF	German	13
Konecny M^[12]^	2016	63	31/6	4/22	NA	BLAF	German	11
Konecny M^[12]^	2016	59	20/11	6/22	NA	Serum	German	13
Wang CH^[13]^	2016	243	79/44	8/112	NA	BLAF	China	8
Ilse P^[14]^	2014	118	48/27	1/42	NA	BLAF	German	12
Dietrich D^[15]^	2013	114	7/51	0/56	NA	Pleural effusion	German	14
Li SF^[16]^	2014	47	10/18	0/9	NA	Pleural effusion	China	10
Ilse P^[17]^	2013	719	138/138	70/373	NA	Pleural effusion	German	13
BLAF: bronchoalveolar lavage fluid; NA: not available; M+/M-: methylation positive/methylation negative; TNM: tumor node metastasis; STROBE: strengthening the Reporting of Observational Studies in Epidemiology.								

**表 2:** 纳入各个研究中真阳性、假阳性、假阴性和真阴性数据分布情况 The TP, FP, FN and TN distribution of the include studies

Author	Time	Sample size	TP	FP	FN	TN
Wang N^[5]^	2018	120	57	1	23	39
Rong QP^[6]^	2018	48	18	2	20	8
Zhang YM^[7]^	2016	277	982	34	32	113
Ren M^[8]^	2017	253	79	10	44	120
Schmidt B^[9]^	2010	523	190	12	91	230
Kneip C^[10]^	2011	343	112	16	76	139
Dietrich D^[11]^	2012	204	78	4	22	100
Konecny M^[12]^	2016	63	31	4	6	22
Konecny M^[12]^	2016	59	20	6	11	22
Wang CH^[13]^	2016	243	79	8	44	112
Ilse P^[14]^	2014	118	48	1	27	42
Dietrich D^[15]^	2013	114	7	0	51	56
Li SF^[16]^	2014	47	10	0	18	9
Ilse P^[17]^	2013	719	138	70	138	373
TP: true positive; FP: false positive; FN: false negative; TN: true negative.						

### 2.2 异质性检验

*SHOX2*基因启动子高甲基化诊断肺癌的敏感性、特异性、阳性预测值、阴性预测值及诊断优势为效应指标行统计学异质性检验，均存在统计学异质性（*P* < 0.05），数据合并均采用随机效应模型。

### 2.3 *Meta*分析

*SHOX2*基因启动子高甲基化诊断肺癌的敏感性为0.75（95%CI: 0.74-0.77）（图 2）、特异性分别为0.89（95%CI: 0.88-0.91）（图 3）；阳性预测值为6.75（4.56-9.99）（图 4），阴性预测值为0.36（0.25-0.52）（图 5）；诊断优势比为23.16（11.34-47.31）（图 6），受试者工作特征曲线（receiver operating characteristic curve, ROC）下面积为0.91（图 7）。根据检测换组织标本的不同，进行了亚组分析，具体见表 3。

**图 2:**
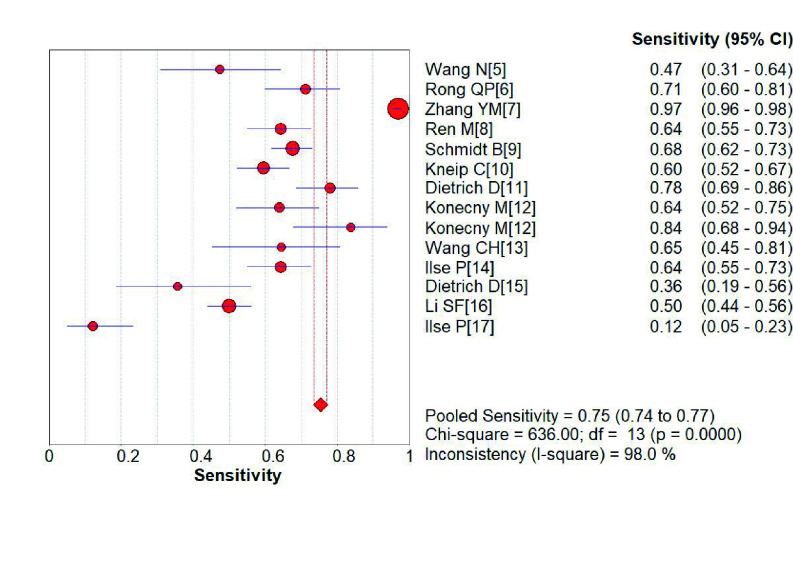
*SHOX2*基因启动子区域异常高甲基化诊断肺癌敏感性的森林图 Forest plot of *SHOX2* gene hypermethylation for lung cancer diagnostic sensitivity

**图 3:**
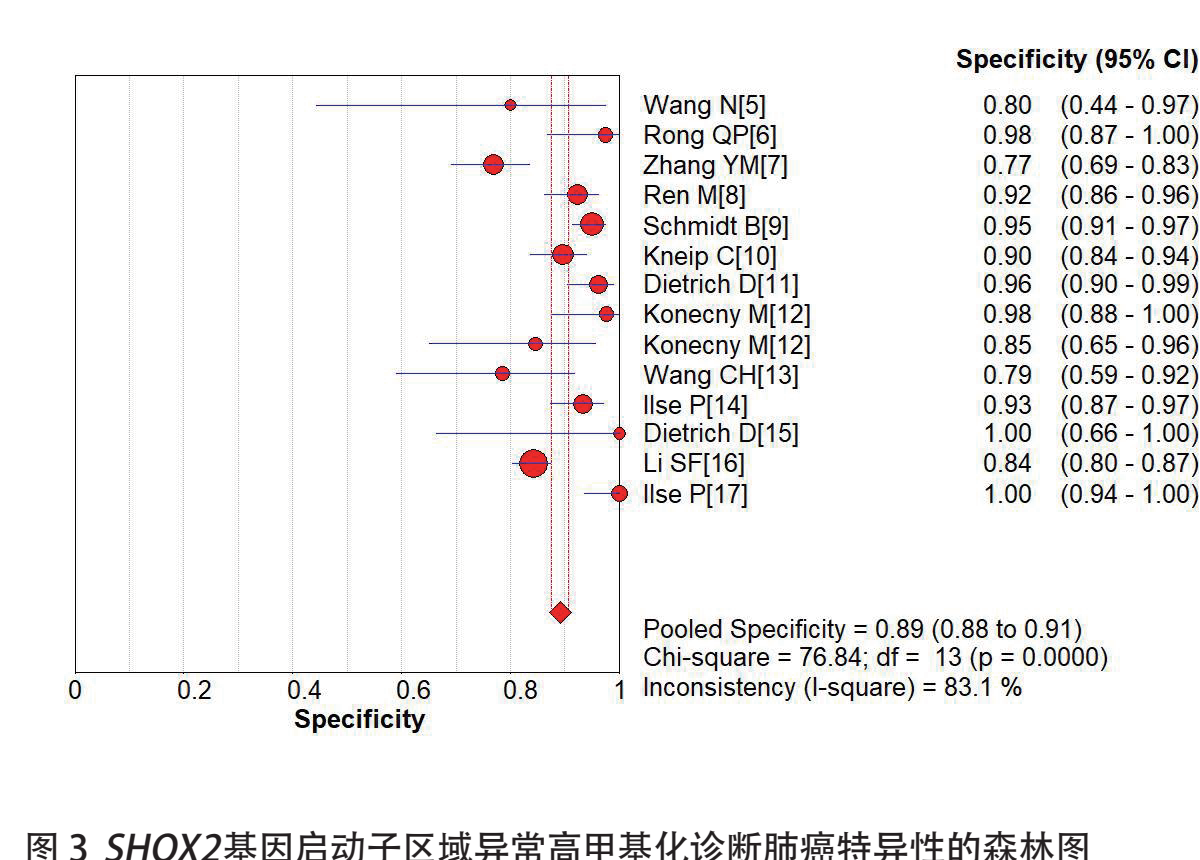
*SHOX2*基因启动子区域异常高甲基化诊断肺癌特异性的森林图 Forest plot of *SHOX2* gene hypermethylation for lung cancer diagnostic specificity

**图 4:**
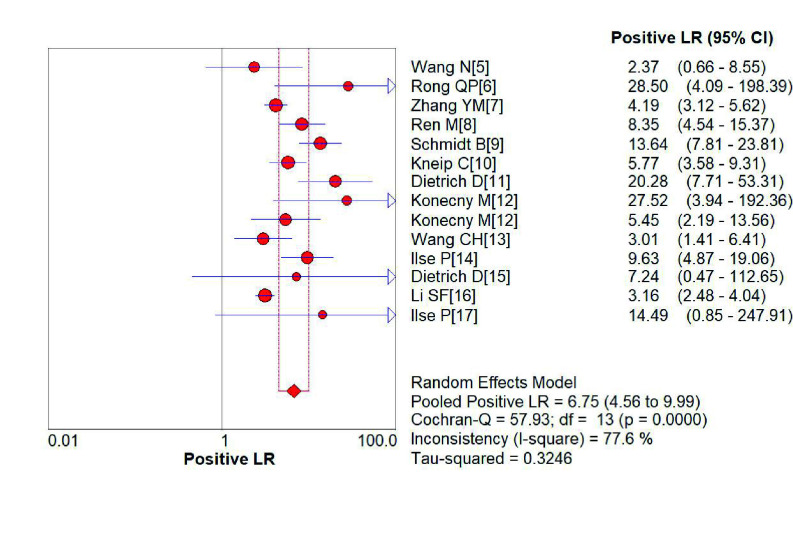
*SHOX2*基因启动子区域异常高甲基化诊断肺阳性预测值性的森林图 Forest plot of *SHOX2* gene hypermethylation for lung cancer diagnostic positive likelihood ratio

**图 5:**
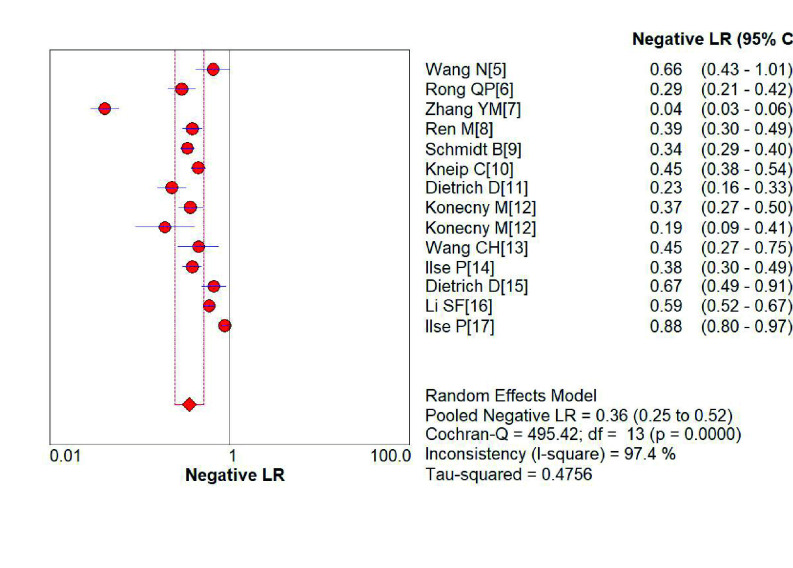
*SHOX2*基因启动子区域异常高甲基化诊断肺阴性预测值性的森林图 Forest plot of *SHOX2* gene hypermethylation for lung cancer diagnostic negative likelihood ratio

**图 6:**
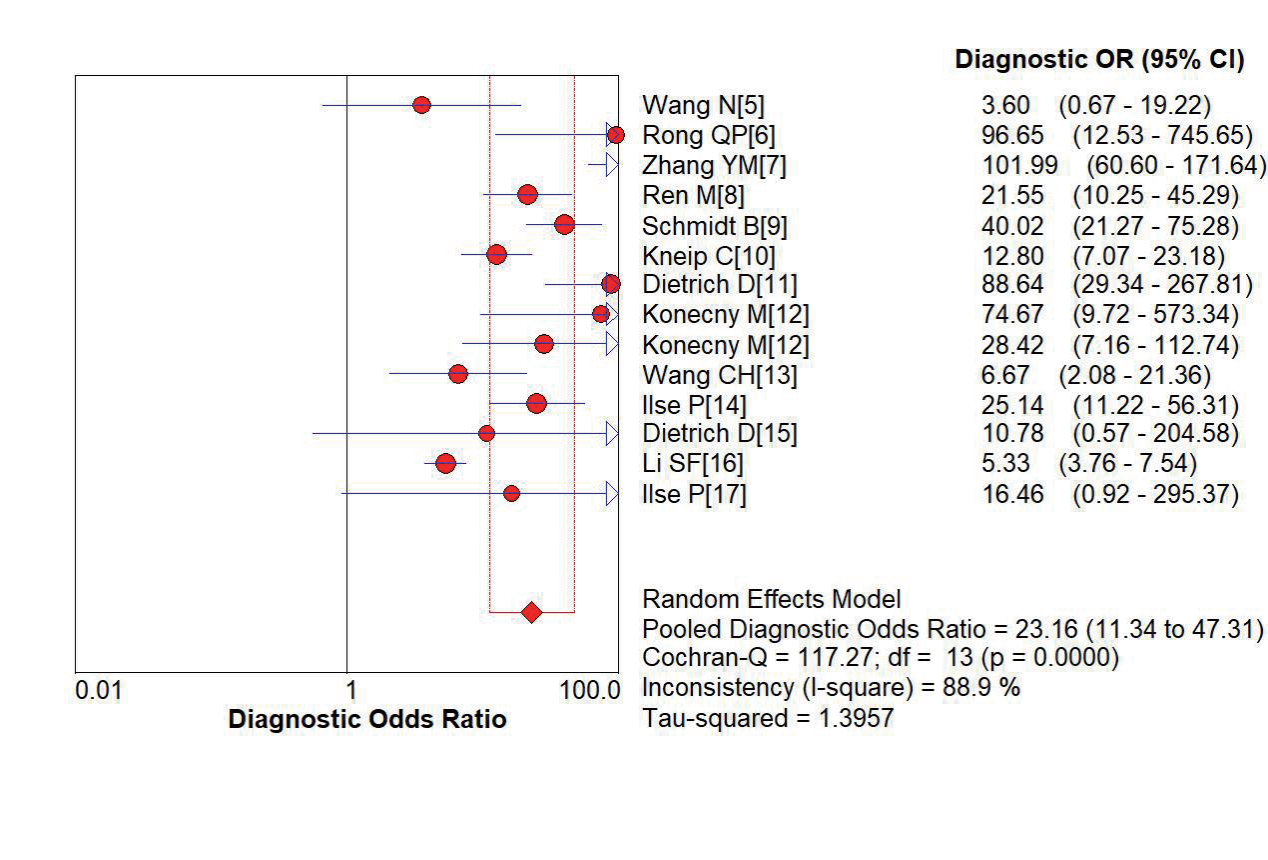
*SHOX2*基因启动子区域异常高甲基化诊断肺诊断优势比的森林图 Forest plot of *SHOX2* gene hypermethylation for lung cancer diagnostic odds ratio

**图 7:**
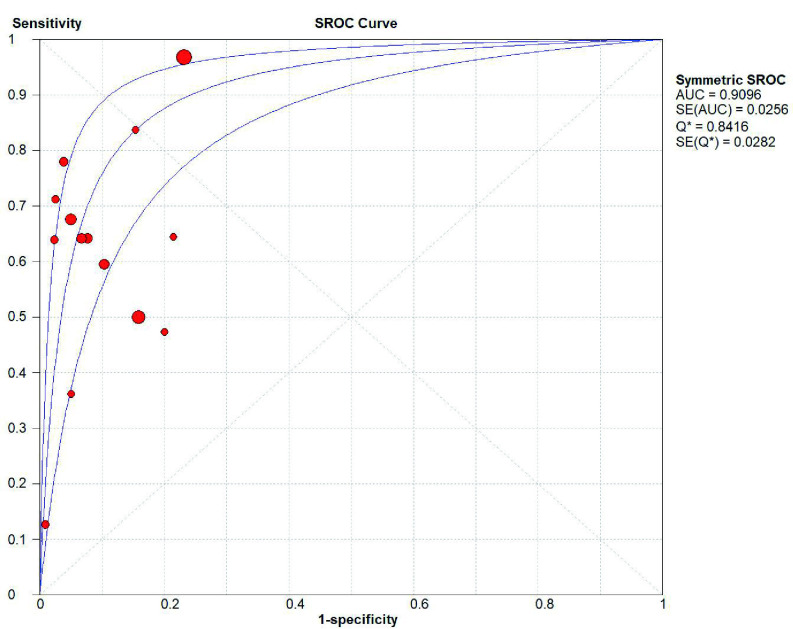
*SHOX2*基因甲基化诊断肺癌的ROC曲线 Summary receiver operating characteristic (SROC) curve of *SHOX2* gene hypermethylation for lung cancer diagnosis

**表 3:** *SHOX2*启动子区域异常高甲基化诊断肺癌价值的亚组分析 Subgroup analysis of *SHOX2* gene hypermethylation for lung cancer

Group	SEN	SPE	+LR	-LR	DOR	AUC
Serum	0.61 (0.56-0.67)	0.89 (0.85-0.93)	4.73 (2.26-9.90)	0.44 (0.33-0.59)	11.37 (4.33-29.84)	0.62
BLAF	0.85 (0.83-0.86)	0.91 (0.89-0.93)	9.12 (5.39-15.46)	0.23 (0.14-0.39)	44.62 (25.31-78.65)	0.94
Pleural effusion	0.43 (0.38-0.48)	0.86 (0.83-0.89)	3.22 (2.53-4.11)	0.71 (0.48-1.04)	5.47 (3.88-7.70)	0.70
SEN: sensitivity; SPE: specificity; +LR: positive likelihood ratio; -LR: negative likelihood ratio; DOR: diagnostic odds ratio; AUC: area under ROC curve; SHOX2; short stature homeobox 2; ROC: receiver operating characteristic.						

## 3 讨论

本研究通过循证医学方法量化分析了*SHOX2*启动子区域甲基化诊断肺癌的价值。结果共纳入了13项研究，显示*SHOX2*基因启动子甲基化诊断肺癌的敏感性为0.75（95%CI: 0.74-0.77）、特异性为0.89（95%CI: 0.88-0.91）；阳性预测值为6.75（4.56-9.99），阴性预测值为0.36（0.25-0.52）；诊断优势比为23.16（11.34-47.31），ROC曲线下面积为0.9。我们的研究结果提示，*SHOX2*基因启动子甲基化在肺癌患者血清、支气管灌洗液和胸腔积液等体液中的发生率均较高，可作为辅助诊断肺癌的生物学标志物。

肺癌是目前世界上最常见的恶性肿瘤^[18]^。迄今，用于肺癌早期诊断和筛查的有效和廉价的方法较少^[19]^。虽然组织学和细胞学检查是诊断肺癌的金标准，但确诊时患者往往处于晚期。因此，迫切需要新的诊断方法来提高早期诊断率，提高确诊率，降低死亡率。*SHOX2*基因甲基化分析被认为是一个具有广泛临床应用前景的诊断方法^[4]^。*SHOX2*基因甲基化检测，与组织学、细胞学检测及影像诊断相结合可提高肺癌的确诊率，并有可能成为早期诊断的有效工具。SHOX2是同源框基因家族的一员，该家族编码含有60个氨基酸残基的蛋白。同源异型盒基因作为转录调节因子广泛存在于无脊椎动物和脊椎动物中^[20]^。近年来，越来越多的研究关注*SHOX2*基因启动子甲基化在肺癌早期诊断中的价值。然而，*SHOX2*基因启动子甲基化在肺癌发生、发展中的生物学意义目前仍未完全阐明。大多数研究^[20, 21]^认为，*SHOX2*基因被证实是多种癌症信号通路的调控子或效应子，可促进肿瘤的发生和发展。*SHOX2*基因甲基化检测在肺癌的发生、发展、转移、耐药和复发过程中起重要作用。*SHOX2*基因甲基化检测联合影像学等其他诊断学方法被认为是筛查和监测肺癌的一种很好的方法，具有较高的敏感性和特异性。

在本研究中，我们对*SHOX2*基因启动子高甲基化作为肺癌辅助诊断生物学标志物的价值进行了量化评价，研究认为*SHOX2*基因启动子诊断肺癌的敏感性和特异性均较高，可作为辅助诊断标志物。但研究本身也存在一定的局限性，首先该*meta*分析仅纳入13项符合要求的原始研究，样本量相对较小，统计学效能不高；其次，只纳入了发表语言为英文和中文的文献，其他语言发表的文献未进行筛选和纳入；第三，诊断的敏感性、特异性及ROC曲线下面积等指标均存在统计学异质性，采用了随机效应模型。因此，后续应及时对发表的文献进行更新检索，并纳入更多符合要求的相关研究，对*SHOX2*启动子区域异常高甲基化诊断肺癌价值进行进一步的评估，提供更为充分有力的循证医学证据。同时，单纯依靠检测*SHOX2*基因启动子甲基化作为肺癌诊断标准，其临床应用价值有限，应结合其他影像学等诊断方法进行综合判断，提高诊断准确性。
